# The Doctor

**Published:** 1874-08

**Authors:** 


*JOURNAL OF MATERIA MEDICA—June*, 1874:

THE DOCTOR.—And who is this man that they call "Doctor?" Strange confession is it to the unity of his order that his individuality is so often merged in his title! The title that graces the most skillful practitioner of medical arts descends through infinite depths and clings to the half-grown boy that unscrews soda water and deals out doses of salts at the pharmacy. The druggists take to the title like ducks to water. The myriad pesthood of patent medicine makers flaunt the title as their claim to the public confidence. Natural bone-setters and eclectics, cancer-curers and homœopaths, old sands-of-life and indian-herbs, equalizer and movement-cure, health-lift, hydropathy, neuropathy, kinesopathy, thomsonianism, clairvoyance, mesmerism and second-sight, with the more cruel types that wallow in the ooze below, and all the leprous and abominable heresies of medicine that prey upon the prejudices and fears of men, flaunt "Doctor" on the red flag of their festering commune.

> "All the grizly legions that troop
> Under the sooty flag of Acheron."

> "Perverse, all monstrous, all prodigious things,
> Abominable, unutterable and worse
> Than fables yet have feigned, or fear conceived
> Gorgons, and Hydras and chimæras dire."

